# Severe Maternal Morbidity Associated with Systemic Lupus Erythematosus Flare in the Second Trimester of Pregnancy

**DOI:** 10.1155/2018/5803479

**Published:** 2018-05-10

**Authors:** Matthew J. Blitz, Adiel Fleischer

**Affiliations:** Division of Maternal-Fetal Medicine, Department of Obstetrics and Gynecology, Long Island Jewish Medical Center, Donald and Barbara Zucker School of Medicine at Hofstra/Northwell, New Hyde Park, NY, USA

## Abstract

Pregnancy in women with systemic lupus erythematosus (SLE) is associated with an increased risk of adverse maternal and fetal outcomes. Here, we present a case of severe maternal morbidity in a 23-year-old primigravida with SLE and secondary Sjögren's syndrome who experienced a life-threatening multisystem flare at 17 weeks of gestational age. She presented to the emergency department complaining of cough with hemoptysis and shortness of breath. She developed hypoxic respiratory failure and was admitted to the intensive care unit. Bronchoscopy confirmed diffuse alveolar hemorrhage. Physical exam and laboratory evaluation were consistent with an active SLE flare, pancytopenia, and new-onset lupus nephritis. After counseling regarding disease severity, poor prognosis, and recommendation for therapy with cytotoxic agents, she agreed to interruption of pregnancy which was achieved by medical induction. Her course was further complicated by thrombotic microangiopathy and generalized tonic-clonic seizures attributable to posterior reversible encephalopathy syndrome versus neuropsychiatric SLE. This case represents one of the most extreme manifestations of lupus disease activity associated with pregnancy that has been reported in the literature and emphasizes the importance of preconception evaluation and counseling and a multidisciplinary management approach in cases with a complex and evolving clinical course.

## 1. Introduction

Systemic lupus erythematosus (SLE) is a chronic autoimmune disease that predominantly affects women of childbearing age. Pregnancy in women with SLE is associated with an increased risk of adverse maternal and fetal outcomes. Here, we present a case of severe maternal morbidity in a young woman with SLE and secondary Sjögren's syndrome who experienced a life-threatening multisystem flare which required a prolonged stay in the intensive care unit and interruption of pregnancy in the second trimester. Although many cases of life-threatening and even fatal maternal complications of SLE have been described in the literature, there are few reported cases in which such a large number of different organ systems are affected, as we describe here. With such an uncertain and complex disease course, clinical management can be very challenging, necessitating a multidisciplinary approach to care.

## 2. Case Presentation

A 23-year-old primigravida at 17 weeks of gestational age presented to the emergency department complaining of worsening cough with intermittent hemoptysis, shortness of breath, and chest pain which had been present for several weeks and exacerbated by light physical activity. Her past medical history was significant for SLE with secondary Sjögren's syndrome diagnosed 6 years before based on findings of arthritis, malar rash, positive antinuclear antibodies (ANA), elevated anti-double-stranded DNA (anti-dsDNA), and the presence of anti-Ro/SSA antibodies. She had no prior surgery and was not taking any medications. She reported no SLE flares in the past few years and had discontinued hydroxychloroquine (HCQ) approximately one year earlier. Her obstetrician referred her for a maternal-fetal medicine consultation in early pregnancy, at which time she acknowledged that she was no longer under the care of a rheumatologist. She did not receive any risk assessment or counseling prior to conception. Two months prior to pregnancy, her serum creatinine was 0.74 mg/dL and her systolic and diastolic blood pressures ranged from 100 to 120 mm Hg and from 70 to 80 mm Hg, respectively. Of note, she was treated with antibiotics for pneumonia three times in the past 14 months and was known to have a recent negative purified protein derivative (PPD) skin test.

**Figure 1:**
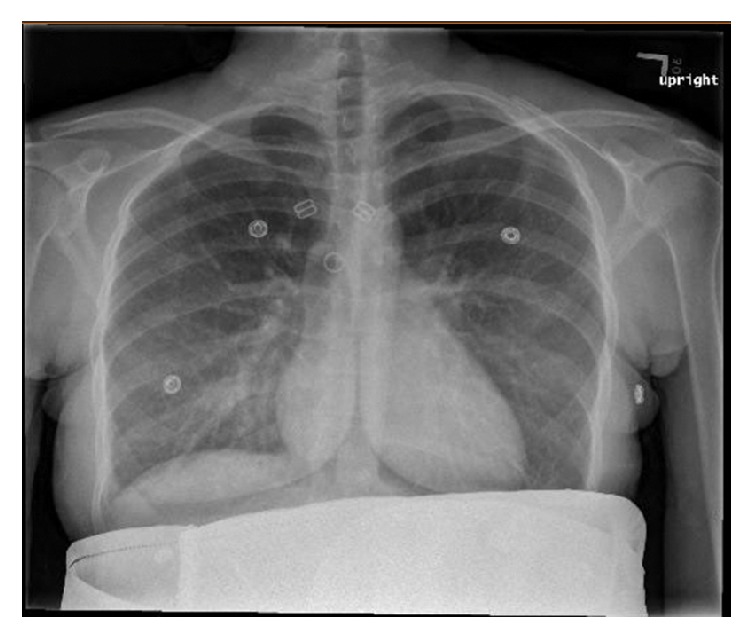
Chest radiograph on the day of admission noted indistinct increased markings and a patchy opacity in the right lower lung.

**Figure 2:**
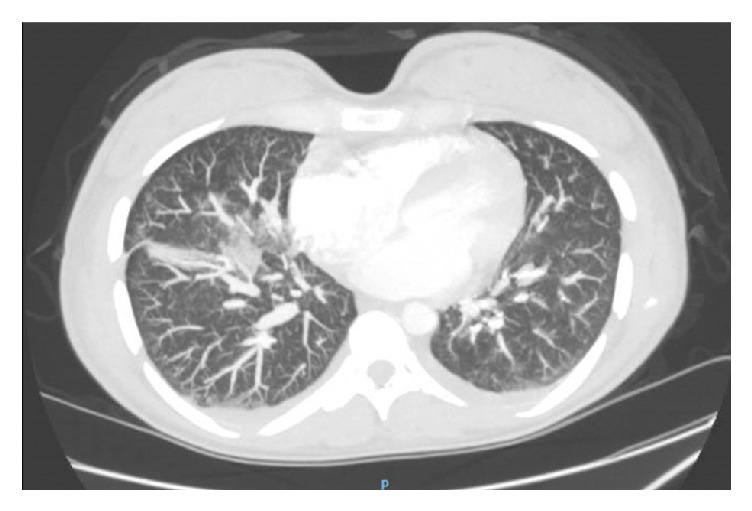
Chest computed tomography angiography (CTA) on the day of admission noting patchy infiltrate in the right middle and lower lung lobes and small bilateral pleural effusions.

Upon arrival to the hospital, she was awake and alert and in no apparent distress. Her heart rate was 116 beats per minute. She was afebrile and normotensive with a respiratory rate of 16 breaths per minute and an oxygen saturation of 100% on room air. Abdominal exam noted a gravid, nontender uterus. Her respiratory effort appeared normal and her lungs were clear to auscultation. A subtle malar rash and diffuse, white ulcerative plaques were visualized on the buccal mucosa and palate. Bilateral lower extremity pitting edema was noted. The physical exam was otherwise unremarkable. Abdominal ultrasound demonstrated an intrauterine pregnancy with a fetal heart rate of 150 beats per minute. Laboratory evaluation was significant for a hemoglobin and hematocrit of 6.1 g/dL and 18.5%, respectively. The white blood cell (WBC) count was 3,300/uL and the platelet count was 101,000/uL. Serum creatinine was 1.04 mg/dL and urine protein/creatinine ratio was 4.7. A 24-hour urine collection contained over 3 grams of protein. Antiphospholipid antibody testing was performed. On hospital day (HD) 1, anticardiolipin IgG and IgM were 24 GPL and 8 MPL, respectively, and repeat testing on HD2 yielded 34 GPL and 11 MPL, respectively (medium or high titer typically defined as >40 GPL or MPL). Anti-beta2-glycoprotein I antibody screen was negative. Lupus anticoagulant assays, which included dilute Russell viper venom time (dRVVT) and silica clotting time (SCT), were negative. An electrocardiogram (EKG) showed sinus tachycardia. Chest radiography featured indistinct increased markings and a patchy opacity in the right lower lung (Figure 1). Chest computed tomography angiography (CTA) found no evidence of pulmonary embolism but did reveal a patchy infiltrate in the right middle and lower lobes with small bilateral pleural effusions and enlarged hilar lymph nodes (Figure 2). After initial evaluation by the obstetrical and emergency medicine teams, consultations were requested with pulmonology, infectious disease, rheumatology, hematology, and nephrology to assist in further management. The decision was made to admit the patient for treatment of suspected community-acquired pneumonia (CAP), new-onset nephrotic syndrome likely secondary to lupus nephritis, and an active SLE flare associated with pancytopenia, malar rash, oral ulcers, and lymphadenopathy.

The patient was started on intravenous ceftriaxone and azithromycin for CAP and HCQ and pulse methylprednisolone for the SLE flare. Thromboprophylaxis was initiated with enoxaparin. She received a transfusion of two units of packed red blood cells (PRBCs) for severe anemia described as normocytic and direct coombs test positive. Indirect bilirubin and lactate dehydrogenase (LDH) were increased, serum haptoglobin was decreased, and a peripheral smear showed a few schistocytes and some spherocytes; these findings were most consistent with autoimmune hemolytic anemia. Overall, her pancytopenia was thought to be multifactorial. In the coming days, there was evidence of continued hemolysis despite steroid administration with a persistently elevated LDH and low serum haptoglobin (<20 mg/dL), as well as repeat peripheral smears demonstrating numerous schistocytes. Of note, a repeat direct coombs test was negative. Collectively, these findings were concerning for other (nonimmune) causes of hemolysis such as microangiopathic hemolytic anemia (MAHA) or thrombotic thrombocytopenic purpura (TTP). A normal ADAMTS13 activity level subsequently ruled out the latter.

**Figure 3:**
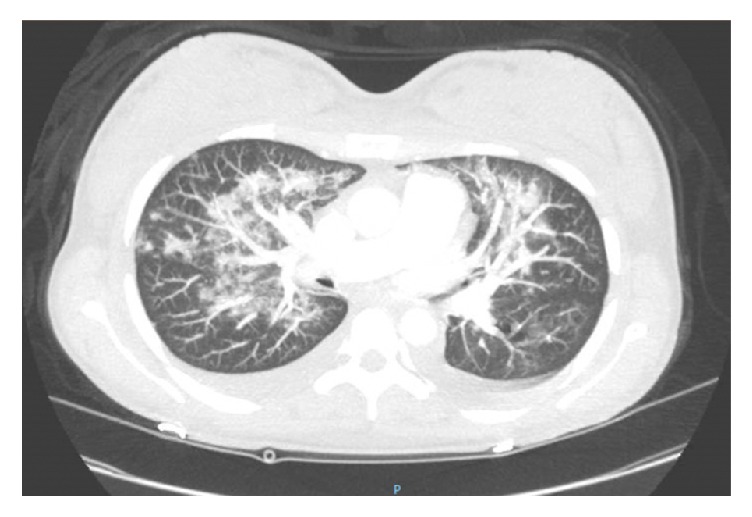
Repeat chest computed tomography angiography (CTA) on hospital day 5 featuring progressive ground-glass opacities bilaterally, which, in the setting of continued hemoptysis, was concerning for diffuse alveolar hemorrhage.

On HD5, the patient was started on supplemental oxygen via nasal cannula after it was noted that her oxygen saturation on room air decreased to below 90%. Over the next few days her oxygen requirements gradually increased to 6 L/min. Pulmonary edema was suspected based on diffuse b-lines on lung ultrasound and repeat chest radiograph noting increased bilateral pleural effusions. All intravenous fluids were discontinued, and furosemide was administered. Improvement in lower extremity edema and good urine output were noted. However, she continued to experience shortness of breath with oxygen saturation as low as 80% on supplemental oxygen. A repeat chest CTA noted progressive ground-glass opacities bilaterally (Figure 3), which, in the setting of continued hemoptysis, increased concern for diffuse alveolar hemorrhage (DAH). On HD8 she was transferred to the intensive care unit for worsening hypoxia despite diuresis, antibiotic therapy, and steroids. Therapeutic plasma exchange (TPE) was performed with a plan for 4 additional sessions. Fiberoptic bronchoscopy with sequential bronchoalveolar lavage (BAL) demonstrated grossly hemorrhagic fluid consistent with DAH.

Given the severity of maternal disease, poor prognosis for the fetus, and recommendation for more aggressive therapy, the patient was counseled for termination of pregnancy. The patient understood that the risk of early-onset preeclampsia and extreme preterm delivery was high even if her pulmonary and renal status were to improve significantly. She was induced and delivered vaginally on HD10. Cyclophosphamide, an alkylating agent associated with gonadal toxicity and teratogenicity, was administered after a suboptimal response to steroids and TPE.

In the first few days after delivery, her blood pressures were persistently elevated (≥140/90 mm Hg) but generally below severe range (160/110 mm Hg). A repeat 24-hour urine collection on HD 14 was found to have a total protein of more than 17 grams. Serum creatinine increased to 1.02 mg/dL. On HD22, a kidney biopsy was performed, which revealed lupus nephritis, diffuse global proliferative type, class IV-G (A), and an active thrombotic microangiopathy (TMA) in several arterioles and glomerular vascular poles. The patient was discharged home on HD25 on prednisone and HCQ for lupus, losartan for hypertension, and furosemide for edema associated with nephrotic syndrome. She was also to continue cyclophosphamide infusions outpatient.

**Figure 4:**
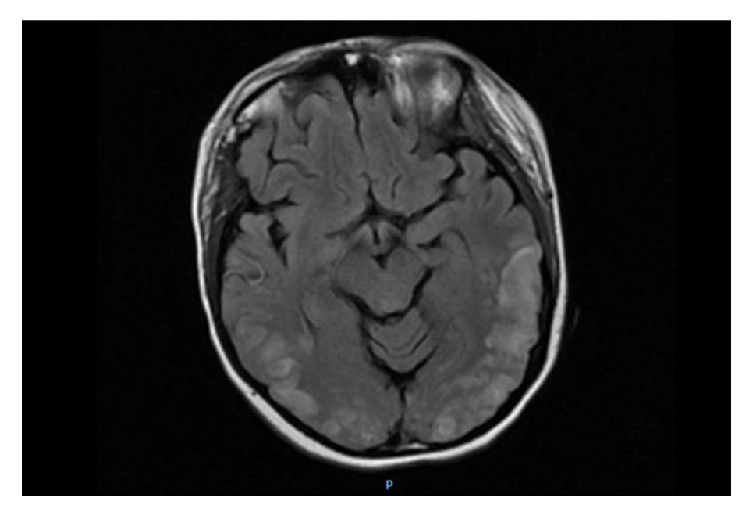
Brain magnetic resonance imaging (MRI) demonstrated symmetrical bilateral hyperintensities on T2-weighted imaging and fluid-attenuated inversion recovery (FLAIR) sequences consistent with cerebrocortical edema.

Less than two weeks after discharge, the patient was readmitted with symptomatic anemia and progressive lower extremity edema. She was also noted to have worsening hypertension (systolic blood pressure 160–200 mm Hg), acute kidney injury (creatinine 1.7 → 2.5 mg/dL), and worsening thrombocytopenia (platelet count 81,000 → 52,000/uL). Serum LDH increased to 1,028 U/L. These findings were suspicious for an SLE flare-induced TMA exacerbation versus atypical hemolytic uremic syndrome (aHUS). Pulse dose steroids were again administered. Nicardipine infusion was started for blood pressure optimization. Approximately one hour after a transfusion of PRBCs was started, the patient was found to have altered mental status. She then experienced a series of generalized tonic-clonic seizures in quick succession without recovery between episodes. This was followed by a postictal state. Lorazepam and levetiracetam were administered. She was intubated and mechanically ventilated for airway protection and transferred to the ICU for status epilepticus. Urgent brain MRI demonstrated symmetrical bilateral hyperintensities on T2-weighted imaging and fluid-attenuated inversion recovery (FLAIR) sequences consistent with cerebrocortical edema (Figure 4); there was no evidence of acute infarct or hemorrhage. Neurology was consulted. Posterior reversible encephalopathy syndrome (PRES) and neuropsychiatric SLE (NPSLE) were highest in the differential diagnosis. Eclampsia was thought less likely at more than 3 weeks postpartum, after delivery of a 17-week fetus. After an inadequate hematologic response to pulse dose steroids, eculizumab, a monoclonal antibody and complement inhibitor, was started. Over the next few days, the platelet count and LDH began improving, suggesting a response to this treatment. The patient received a total of 5 units of PRBCs and 3 doses of eculizumab and was successfully extubated on HD5 after readmission. Repeat antiphospholipid antibody testing was performed. Anticardiolipin IgG and IgM decreased to 4 GPL and 3 MPL, respectively. Anti-beta2-glycoprotein I antibody screen and lupus anticoagulant functional assays remained negative. She was discharged home on HD13 on labetalol and amlodipine for blood pressure control, HCQ and prednisone for lupus, levetiracetam for seizure prophylaxis, and cefuroxime for meningitis prophylaxis given that eculizumab increases susceptibility to Neisseria infection.

Over the next several months, she experienced continued seizure activity and frequent headaches which required intermittent hospitalization.

## 3. Discussion

It is well established that pregnant women with SLE have an increased risk of adverse maternal and fetal outcomes, including, among others, preeclampsia, venous thromboembolism, infection, unplanned cesarean delivery, fetal growth restriction, preterm birth, and fetal loss [1]. In addition, maternal mortality may be 20-fold higher in women with SLE [2]. Active disease and lupus nephritis are nearly always present in such cases, and the two major causes of death are complications from lupus disease activity and opportunistic infection [3].

Based on the numerous risks associated with pregnancy, it is recommended that women with SLE have a preconception evaluation and multidisciplinary management with maternal-fetal medicine and rheumatology during pregnancy. Active SLE at the time of conception is a predictor of adverse outcomes; it is suggested that the disease be quiescent for six months prior to attempting pregnancy [4]. Laboratory testing should, at a minimum, include antiphospholipid antibodies (lupus anticoagulant, IgG and IgM anticardiolipin antibodies, IgG and IgM anti-beta2-glycoprotein I antibodies), anti-Ro/SSA and anti-La/SSB antibodies, and an assessment of renal function (creatinine, spot urine protein/creatinine ratio). Women who have anti-Ro/SSA and anti-La/SSB antibodies should have intensive fetal surveillance for heart block with weekly pulsed-Doppler fetal echocardiography (to measure the mechanical PR interval) starting at 16–18 weeks and continuing through 26–28 weeks of pregnancy. Ideally, all women with SLE should be on HCQ and low-dose aspirin during pregnancy, unless otherwise contraindicated. Women who continue HCQ during pregnancy have fewer disease flares, improved outcomes, and, in mothers with anti-Ro/SSA and anti-LA/SSB antibodies, a reduced risk of fetal heart block [5]. Low-dose aspirin started at 12–16 weeks of gestation reduces the risk of preeclampsia and fetal growth restriction [6]. Discontinuation of medications used to control disease activity increases the risk of flares and associated pregnancy complications. Serial ultrasound examinations should be performed to assess fetal growth and antepartum fetal surveillance should be initiated in the third trimester.

Renal involvement is common in patients with SLE and may be suspected in the presence of proteinuria or an elevated serum creatinine. Lupus nephritis is diagnosed and classified based on histopathologic findings on renal biopsy [7]. Most patients with this condition have an immune complex-mediated glomerular disease. The most common and most severe form of lupus nephritis is class IV, defined histologically as involvement of more than 50 percent of glomeruli on light microscopy [8]. Class IV disease is further subclassified based on whether affected glomeruli are segmentally (S) or globally (G) affected and whether the inflammatory activity of the lesions is active (A), chronic (C), or both (A/C). Hypertension and nephrotic syndrome, consisting of heavy proteinuria, hypoalbuminemia, and peripheral edema, are often seen in active class IV disease and patients characteristically have low complement levels (C3) and elevated anti-dsDNA levels.

Involvement of the renal vasculature in cases of lupus nephritis is a poor prognostic sign. In thrombotic microangiopathy (TMA), damage to the endothelial cells of small arterioles and capillaries results in microvascular thrombosis. Nearly all TMAs cause microangiopathic hemolytic anemia (MAHA) and thrombocytopenia. MAHA results from intravascular fragmentation of red blood cells, which produces schistocytes on peripheral blood smear.

Alveolar hemorrhage is a rare, life-threatening complication which occurs in approximately 0.5–3.7% of nonpregnant SLE patients [9, 10] and has also been reported during pregnancy in a limited number of case reports and case series [11–13]. This is typically associated with active class III or IV lupus nephritis and increased disease activity. Microvascular injury is thought to result from immune complex deposition in the alveolar wall with induction of apoptosis [14, 15]. The mortality rate for SLE patients with DAH may exceed 50% [9, 16]. It is important to recognize that hemoptysis is reported in less than two-thirds of such cases [17]. More frequent signs and symptoms are hypoxemia, dyspnea, cough, and fever. Thus, DAH can easily be mistaken for pneumonia or pulmonary edema [18]. In patients with lupus nephritis, clinical suspicion for DAH should be increased in the presence of acute pulmonary symptoms, new radiographic infiltrates, and a falling hematocrit, even in the absence of hemoptysis. The diagnosis is established by flexible bronchoscopy with sequential BAL. Characteristically, lavage aliquots are progressively more hemorrhagic and Prussian blue staining reveals hemosiderin-containing macrophages [19, 20]. High clinical suspicion, early diagnosis, and aggressive therapy with glucocorticoids and cyclophosphamide are the cornerstones of DAH management in patients with SLE. Ventilatory support and blood transfusion should be offered as necessary. Although it is uncertain whether it improves survival, plasma exchange therapy is frequently offered to remove circulating immune complexes from the blood, especially in cases where there is an inadequate clinical response to cyclophosphamide [21].

Posterior reversible encephalopathy syndrome (PRES), characterized by headache, visual disturbances, confusion, and seizures, is an underrecognized condition associated with SLE [22]. Pregnancy-related PRES in women* without* SLE occurs most frequently in conjunction with preeclampsia-eclampsia [23]. Disruption of the blood brain barrier secondary to endothelial dysfunction results in vasogenic edema, often in the parietooccipital regions; this produces a characteristic magnetic resonance imaging (MRI) finding of hyperintense signal in affected areas on T2-weighted imaging [24, 25]. At the time of presentation, most SLE patients diagnosed with PRES have hypertension, lupus nephritis, and active disease with multiple complications [26, 27].

Although it is one of the most prevalent complications of lupus, likely affecting the majority of patients at some time in their disease course, NPSLE is arguably the most poorly understood [28, 29]. NPSLE associated with pregnancy has been addressed in scant case reports and case series [30, 31]. Nervous system involvement can produce a complex array of neurological, psychiatric, and behavioral manifestations that increase morbidity and mortality. The American College of Rheumatology (ACR) has developed a standardized nomenclature system which defines 19 NPSLE syndromes involving the central and peripheral nervous systems [32]. Cognitive dysfunction, headaches, and psychiatric disorders are some of the most common NPSLE syndromes [29, 33]. In all cases, NPSLE is a diagnosis of exclusion; all other possible etiologies of the observed neuropsychiatric symptoms must be considered and excluded, including electrolyte abnormalities, infection, renal failure, and drug effects. In the absence of a gold standard diagnostic test, this may represent a significant clinical challenge, especially in pregnancy and the postpartum period, where pregnancy-specific conditions such as preeclampsia-eclampsia may produce the same symptoms. The pathogenesis of NPSLE is multifactorial and involves inflammatory cytokines, autoantibodies, and immune complexes that contribute to vasculopathic, cytotoxic, and autoantibody-mediated neuronal dysfunction [34]. Management remains empirical without any standardized, evidence-based approach to treatment [35].

Antiphospholipid syndrome (APS) is an autoimmune disorder characterized by vascular thromboses and/or pregnancy morbidity in the presence of persistent antiphospholipid antibodies. A small subset of patients with APS (<1%) develop multiple organ failure secondary to widespread thrombotic disease, a condition referred to as catastrophic APS (CAPS) which has a mortality rate of up to 50% [36]. A definitive diagnosis requires simultaneous involvement of 3 or more organs and histopathologic confirmation in at least one organ or tissue. In the case described here, the patient did not meet criteria for APS. She did have anticardiolipin antibodies but at titers below the threshold to meet APS laboratory criteria (>40 GPL or MPL). However, the clinical similarities with CAPS are worth noting. Although it is possible that serial laboratory evaluation during her first hospital admission would have yielded an anticardiolipin IgG titer > 40 GPL, the anticardiolipin results during her second admission demonstrate that they were not persistent and, therefore, not consistent with APS or CAPS.

Termination of pregnancy in the second trimester, prior to viability, to preserve the health of the mother in cases of severe SLE disease activity is infrequent and usually performed to facilitate more aggressive treatment. Although the physiologic changes of pregnancy, including an increase in glomerular filtration rate and renal plasma flow, may worsen preexisting renal disease, there is not sufficient evidence to suggest that interruption of pregnancy, in and of itself, has a beneficial effect on the resolution of an acute disease flare. However, it is theoretically possible that a rapid decline in pregnancy hormone levels, particularly estrogen, may be advantageous [37]. Immunosuppressive medications used to treat SLE, such as cyclophosphamide, are known to cross the placenta and have teratogenic effects. In addition, this particular medication has been associated with premature and irreversible ovarian failure. It should only be offered, after appropriate counseling, in cases where no alternative therapy is available.

In the case presented here, the patient did not have many of the known prepregnancy predictors of adverse pregnancy outcomes. Specifically, she did not have any recent disease exacerbations, antiphospholipid antibodies, thrombocytopenia, prior lupus nephritis, or prior use of antihypertensive medications [1, 38]. However, as a primigravida, she was at increased risk for flare during pregnancy [39]. In addition, her discontinuation of HCQ prior to pregnancy, lack of preconception counseling, and lapse in rheumatologic care placed her at increased risk of pregnancy morbidity.

In summary, SLE disease flares can occur at any time during pregnancy and with unpredictable severity. All women with a diagnosis of SLE should be offered a preconception evaluation and consultation, with a goal of reducing adverse pregnancy outcomes through health education, risk assessment, and appropriate interventions. In cases with a complex and evolving clinical course, the importance of a collaborative and multidisciplinary management approach must be emphasized. Furthermore, it is essential that management occur at a tertiary referral center with experience managing critically ill high-risk obstetric patients.
